# Electrical Properties of Multi-Pyrene/Porphyrin-Dendrimers

**DOI:** 10.3390/molecules200917533

**Published:** 2015-09-22

**Authors:** Mark Euguenii Martínez-Klimov, Ulises Organista-Mateos, Andrés Borja-Miranda, Margarita Rivera, Oscar Amelines-Sarria, Marcos Martínez-García

**Affiliations:** 1 Instituto de Química, Universidad Nacional Autónoma de Mexico, Ciudad Universitaria, Circuito Exterior, Coyoacán, C.P. Mexico D.F. 04510, Mexico; E-Mails: klimov@comunidad.unam.mx (M.E.M.-K.); ulises_orgma@outlook.com (U.O.-M.); borja.m.a@hotmail.com (A.B.-M.); 2 Instituto de Física, Universidad Nacional Autónoma de México, Ciudad Universitaria, Circuito Exterior, Coyoacán, C.P. Mexico D.F. 04510, Mexico; E-Mails: mrivera@fisica.unam.mx (M.R.); oscar.amelines@fisica.unam.mx (O.A.-S.)

**Keywords:** dendrimers, pyrene, PAMAM, porphyrin

## Abstract

Dendrimers bearing pyrene donor groups have been obtained and act as efficient light-harvesting antennae capable of transferring light energy through space from their periphery to their core. The light-harvesting ability increases with each generation due to an increase in the number of peripheral pyrenes. In order to evaluate the photovoltaic properties of the compounds, thermal evaporated thin films were produced and the voltage response in the presence of visible light was obtained. The energy transfer efficiency was found to be almost quantitative for the first and second generations. The dendrimers have the potential to become integral components of molecular photonic devices.

## 1. Introduction

**Scheme 1:**
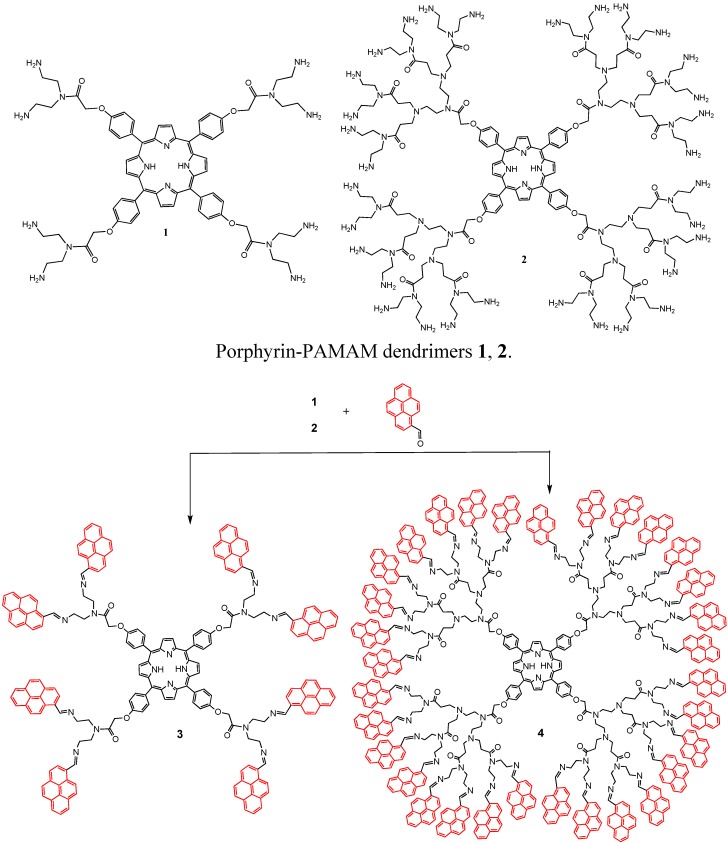
Chemical structures of the pyrene-dendrimers.

Porphyrin-core dendrimer systems have been of much interest lately due to the variety of terminal substituent groups on the photoactive core, which can modulate physico-chemical properties [1,2,3,4]. These molecules have been studied for a broad range of applications, including the solubilization of insoluble molecules, protection from the surrounding environment [3], shape-selective catalysis [5], light-emitting diodes [6], photodynamic therapy [7], chemical sensors [8], optical limiters [9] and so on. In recent years, an increased number of dendrimer-decorated porphyrins have been reported, as well as a large number of different porphyrins bearing pendant linear oligopyrene arms [10], which opens the possibility to join them with PAMAM dendrimers. PAMAM dendrimers (poly(amidoamine)s) are a recent class of dendrimers that are produced commercially, which possess an established molecular composition with different terminal functional groups. First synthesized and investigated by Tomalia [1], these macromolecules could present new interesting properties with expanded areas of application when the dendrimers are modified with fluorescent units. Here, we propose to use donor and acceptor (DA) compounds with anchoring groups of different lengths as a potential approach to construct ordered DA architectures with different numbers of pyrene groups. Porphyrins and pyrenes are widely studied and have been confirmed to be an excellent combination as a donor-acceptor pair [11,12,13,14,15,16,17,18]. Porphyrins are highly conjugated macrocycles that absorb light over a wide wavelength range in the visible and UV regions and have high electron-accepting abilities. The activity in DA systems can be altered by changing the peripheral substituents of macrocycles [19]. We have previously reported the synthesis of porphyrin-PAMAM-dendrimers with fluorenyl chromophores in the periphery [20]. In this article, we describe the peripheral modification of porphyrin-PAMAM dendrimers with pyrenyl chromophores (Scheme 1). In addition, we present the ability to control and manipulate energy-transfer processes in synthetic systems, where the porphyrin ring embedded at the core of the dendrimer was used as an electrochemically active moiety to study the accessibility of the dendrimer core and the pyrene through electron-transfer reactions between an electron donor and an electron acceptor.

## 2. Results and Discussion

Previously, we reported the synthesis of porphyrin-PAMAM dendrimers of first and second generation [20]. The Dendrimers **1** and **2** were obtained with diethylenetriamine in a mixture of methanol and toluene 1:1 at reflux for 24 h.

To modify the properties of the porphyrin-PAMAM dendrimers and obtain good DA systems, the dendrimers were joined 8 and 32 pyrene groups, for this, the 1-pyrene carboxaldehyde moiety was attached to the porphyrin-PAMAM dendrimers in methanol/benzene 1:1 at reflux, to obtain the compounds **3** and **4** (Scheme 1).

### 2.1. Optical Properties in Solution

**Figure 1:**
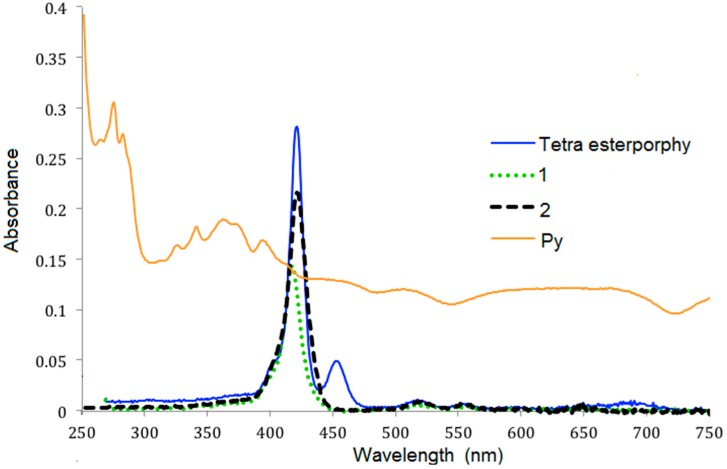
Absorption spectra of 1-pyrenecarboxaldehyde, tetra esterporphyrin and dendrimers **1**, **2** in CH_2_Cl_2_ at room temperature at the concentration of 3.0 × 10^−6^ M.

The absorption spectra of 1-pyrenecarboxaldehyde, the tetra ester-porphyrin and dendrimers **1** and **2** in CH_2_Cl_2_ are presented in Figure 1. The absorption spectra of tetra esterporphyrin and dendrimers **1** and **2** contain the characteristic peaks of the porphyrin. A Soret band at 415–421 nm and the Q bands between 456 to 685 nm, The λ_max_ of absorption of the first generation dendrimer **1** was bathocromic shifted fom 415 nm to 421 nm for the second generation **2**, for the 1-pyrenecarboxaldehyde were observed two broad bands at 276 and 341 nm. For the tetra esterporphyrin near to the Soret band has small shoulder at 454 nm, which could be assigned to the y-polarized transition, while the band at 421 is assigned as the Qx and/or Qy transition [21].

**Figure 2:**
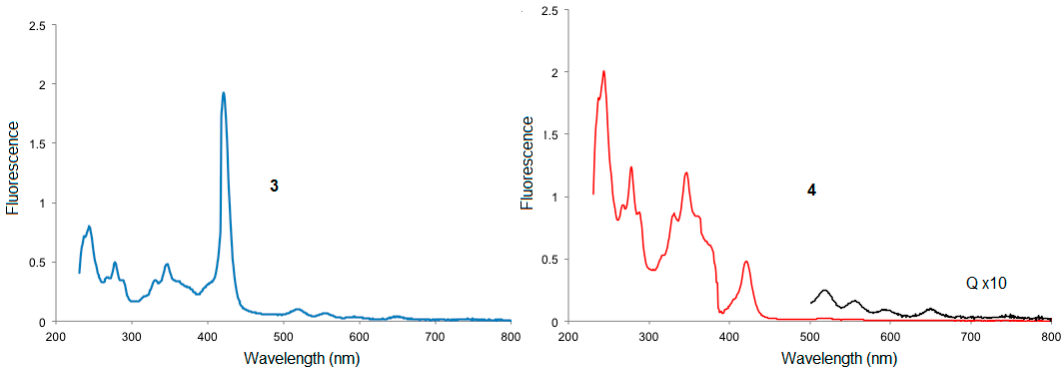
Absorption spectra of dendrimers **3** and **4** in CH_2_Cl_2_ at room temperature at the concentration of 3.0 × 10^−6^ M.

For the dendrimers with pyrene in the periphery **3** and **4** were observed signals due to the aromatic rings at the pyrene fragment, the Soret and the Q bands. For the second generation dendrimer **4**, the Soret and Q bands were diminished; this could be due to the presence of 32 pyrene moieties in the structure of the dendrimer (Figure 2).

**Figure 3:**
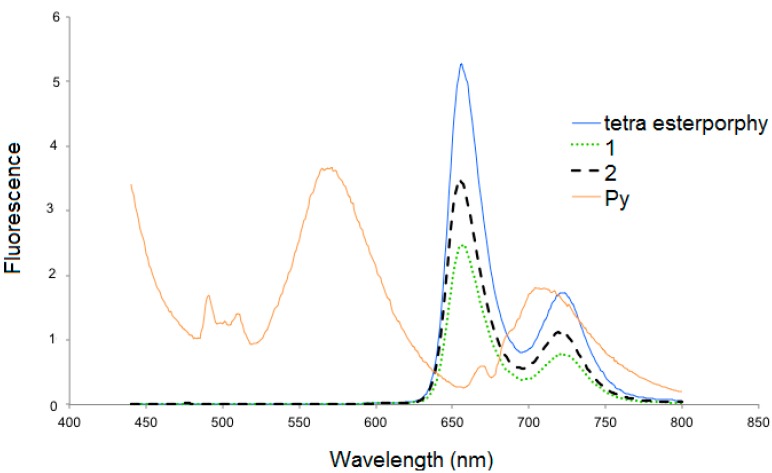
Fluorescence spectra of 1-pyrenecarboxaldehyde tetra esterporphyrin and dendrimers **1**, **2** in CH_2_Cl_2_ at room temperature at the concentration of 3.0 × 10^−6^ M.

Dendrimers **1** and **2**, after excitation at 420 nm, showed one broad band at 650 nm and a small shoulder at 719 nm, the intensity of which was higher for the tetra esterporphyrine dendrimer than for the second generation dendrimer **2** (Figure 3). The 1-pyrenecarboxaldehyde was excited at 275 nm and two peaks were observed at 566 and at 703 nm.

**Figure 4:**
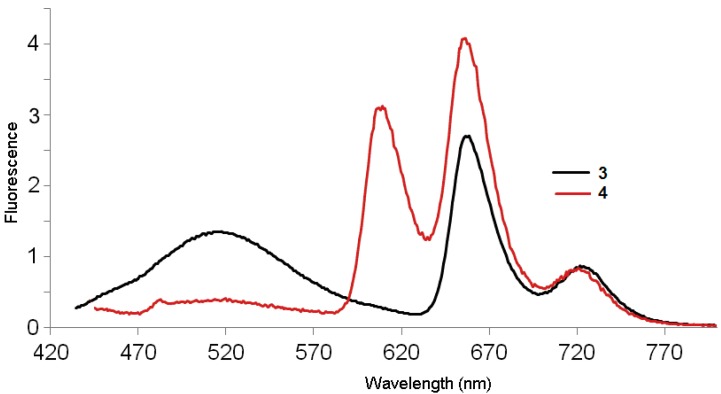
Fluorescence spectra of dendrimers **3** and **4** in CH_2_Cl_2_ at room temperature at the concentration of 3.0 × 10^−6^ M (λ_ext._ 420 nm).

When dendrimers **3** and **4** are excited at 420 nm in the Soret band, the emission spectra for the first generation dendrimer **3** with 8 pyrene moieties revealed three strong bands at 520, 654 and 727 nm (Figure 4). The emission spectrum of compound **3** showed a broad, long-wavelength band (450–550 nm), which is diagnostic of the intramolecular excimer emission (50%) [22,23,24]. The broad fluorescence peak observed at 520 nm is well-known for the formation of π-π interaction between adjacent parallel-oriented pyrene chromophores, the excimer emission suggesting that the first generation favors the parallel arrangement between peripheral pyrene rings, and is a typical *J*-band corresponding to *J*-type aggregates, which could be slipped face-to-face packing between the pyrenes [25]. As suggested by the previously described modeling studies, these results indicate that the pyrene groups are in electronic communication with one another.

For dendrimer **4**, three peaks can be observed: one at 605 nm, a strong red fluorescence with a peak maximum at 653 nm and a weak shoulder at 727 nm (Figure 4) and almost no residual emission of the pyrene. Thus, there is apparently a good energy transfer between pyrene and porphyrin. The intensity of the excimer emission from **4** (b) (from 460–550 nm) decreased with the presence of 32 pyrene units, the excimer fluorescence of second generation dendrimer has even lower intensity than that of the first generation. This may be due to the fact that peripheral chromophores in the second generation is more sterically crowded, which in turn prevents the pyrene rings from parallel arrangement and manifest the strong effect of dendritic architectures on the fluorescence spectra [26].

Table 1 shows the absorption spectral data of compounds **3** and **4** in CH_2_Cl_2_ at 24 °C at the concentration of 3.0 × 10^−6^ M, where the molar extinction coefficient (ε) for dendrimers **3** and **4** was slightly red shifted from 422 to 424 nm (solvatochromism) with an increase in ε. These results could be a consequence of charge-transfer between the donor-acceptor pyrene moiety and the porphyrin core.

**Table 1:** Electronic absorption spectral profiles of dendrimers in CH_2_Cl_2_.

Sample	Pyrene_max_ (nm)	B_max_ (nm)	Q_max_ (nm)	ε × 10^−5^ (M^−1^∙cm^−1^)
**3**	243, 278, 346	422	519, 577, 591, 654	2.7296
**4**	244, 280, 356	424	522, 573, 590, 656	2.8227

### 2.2. Film Properties

**Figure 5:**
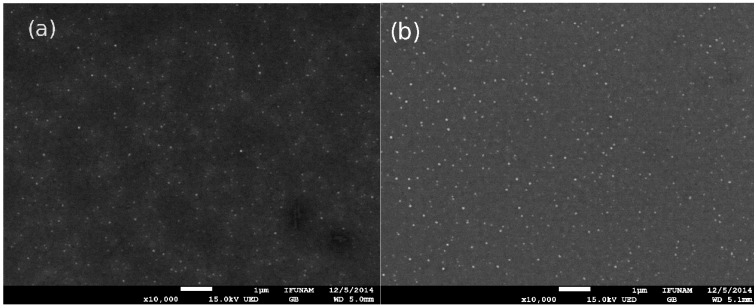
Scanning electron microscope (SEM) images of films obtained from compounds (**a**) **3** and (**b**) **4**. Magnifications are 10,000× in both cases.

In Figure 5, SEM images of the first (a) **3** and second (b) **4** generation dendrimers are shown. In both cases, the surface coverage of the substrate is complete. In addition, SEM images indicate the presence of very regular films with smooth surfaces. It is worth noticing that some bright spots can be seen, which suggest the formation of very small aggregates.

**Figure 6:**
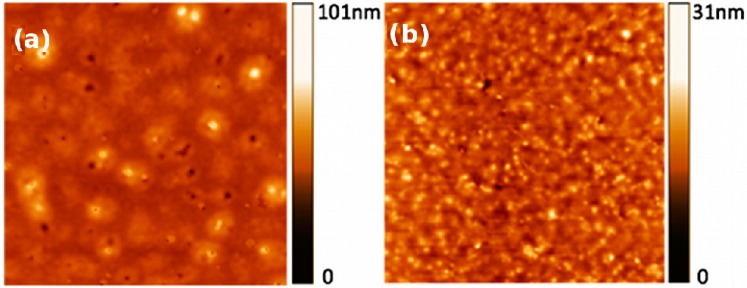
Atomic force microscopy (AFM) images showing the surface morphology of films (**a**) **3** and (**b**) **4**. Image sizes are 10 × 10 μm.

Figure 6 shows the surface morphology of the films obtained with AFM. By measuring the height of the films, thicknesses of 45 nm and 48 nm were obtained for compounds **3** and **4**, respectively. Figure 6a, corresponding to the **3** film, showed a very flat and homogeneous surface. The film exhibited few defects as grains or pores irregularly distributed on the film surface. The roughness value of this surface was 6.67 nm. On the other hand, Figure 6b, corresponding to the **4** film, showed a granular texture and a roughness value of 3.17 nm. In both cases, both compounds produced very homogeneous films.

### 2.3. Photovoltaic Properties

**Figure 7:**
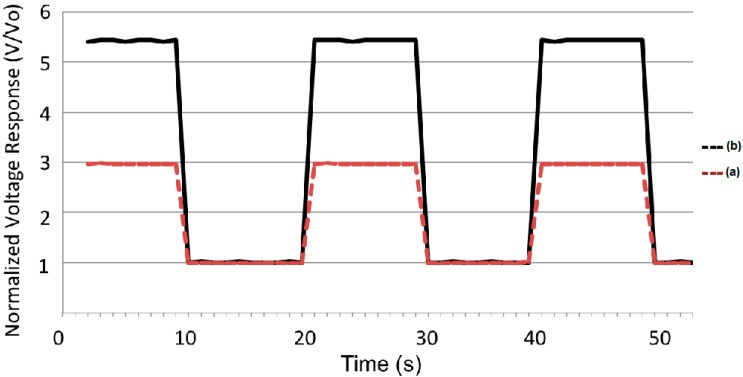
Photovoltaic response of films (a) **3** and (b) **4**.

The photovoltaic response of the films is shown in Figure 7. When the fixed light flux was imposed, a very steady and reproducible conversion was observed. In both cases, the voltage response was normalized in order to compare the voltage conversion for both compounds. From these plots, the relative magnitudes of steady-state voltage can be followed for hours without any appreciable loss in both cases.

In Figure 5 it can be clearly seen that compound **4** exhibits a larger photovoltaic response in comparison with compound **3**. It is worth mentioning that the voltage change for the **4** film was 5.43 times larger in the presence of light with respect to the base voltage value. In contrast, the **3** film only showed an increase of 2.97 times the base voltage value. From these numbers, it is clear that these dendrimers exhibit a good photovoltaic efficiency in comparison with other systems [16]. The difference in response between both molecules can be attributed to better molecular stacking within the films, due to the size and geometry of the molecules, which promotes a larger recombination of charge carriers.

## 3. Experimental Section

### 3.1. General Information

The UV-vis absorption spectra were obtained at room temperature with a Shimadzu 2401 PC spectrophotometer, while a Perkin-Elmer LS-50 spectrofluorimeter was used for the fluorescence spectra. The excitation wavelengths used for the emission properties are reported in the text. The samples for photoelectric measurements were prepared on glass plates covered with a semitransparent layer of indium tin oxide (ITO) with a sheet resistance of 10 ohms/sq. The substrates were cleaned and nitrogen dried before each evaporation. Thin films of compounds **3** and **4** were deposited by using a NORM vacuum thermal evaporator VCM 600 working at 10^−6^ mbar pressure. The morphological properties of each film were evaluated with a JEOL FE7800F scanning electron microscope (SEM) working at 15 KV and the gentle beam method, in order to minimize destruction and decrease charging on the samples. In addition, atomic force microscopy (AFM) images were obtained with a JEOL JSPM4210 instrument in the tapping mode to determine the morphology, thickness and roughness of the films.

The photo-response of the films was evaluated with a homemade device to detect the voltage change through a follower circuit. The photovoltaic response was induced with an Oriel LCS-100 solar simulator with an AM1.5G filter. The voltage values were measured with a Keithley 2400 digital SourceMeter instrument for compounds **3** and **4** under the same experimental conditions at room temperature. In addition, both surfaces were exposed to 10 s intervals of light/dark in order to determine reproducibility and time response.

### 3.2. Synthesis of Dendrimers ***3*** and ***4*** with Pyrene in the Periphery

To a solution of dendrimers **1** and **2** (0.5 mmol) in methanol (15 mL), 1-pyrene carboxaldehyde (0.5 mmol) in methanol (15 mL) and benzene (15 mL) were added, the mixture was stirred in a nitrogen atmosphere at 45–50 °C for 3 days. The methanol and benzene were removed *in vacuo*. The residue was washed several times with methanol first and then with CH_2_Cl_2_ to obtain the desired products **6** and **7**.

*Compound* **3**. Purple solid in 85% yield. m.p: ˃300 °C. FTIR (pellet, KBr, cm^−1^): 3297, 2938, 1651, 1545, 1470, 1348, 1290, 1235, 1176, 1120, 797, 731, 587. UV-vis (CH_2_Cl_2_): λ_max_ 243, 278, 346, 422, 519, 577, 591, 654. ^1^H-NMR (300 MHz, CDCl_3_) δ_H_: −2.79 (br, 2H, NH), 1.90–1.96 (m, 16H, CH_2_-N), 3.30–3.54 (m, 16H, CH_2_-N), 4.90 (s, 8H, CH_2_-O), 7.41 (br, 8H, Ar), 7.57–8.40 (br, 72H, pyrene, Ar-O), 8.08–8.11 (br, 8H, Ar), 8.85 (br, 8H, pirrol). ^13^C-NMR (75 MHz, CDCl_3_) δ_C_: 38.8 (CH_2_-NH_2_), 40.4 (CH_2_-N), 41.6 (CH_2_-N), 43.4 (CH_2_-N), 53.7 (CH_2_-N), 57.8 (CH_2_-N), 58.4 (CH_2_-O), 69.1 (CH_2_-O) 108.6 (Ar), 115.0 (Ar_ipso_), 137.3 (pyrrol), 164.6 (C=O), 171.9 (C=O), 175.2 (C=O), 175.6 (C=O). MS MALDI TOF *m*/*z*: 2947 (M^+^). Anal. Calcd for C_204_H_146_N_16_O_8_. C 83.07, H 4.99, N 7.60%. Found: C, 83.06; H, 5.01; N 7.58%.

*Compound* **4**. Purple solid in 82% yield. (film, cm^−1^): 3257, 3074, 1644, 1547, 1433, 1368, 1293, 1239, 1178, 1043, 998, 642, 597. UV-vis (CH_2_Cl_2_): λ_max_ 284, 424, 512. ^1^H-NMR (300 MHz, CDCl_3_) δ_H_: −2.86 (br, 2H, NH), 2.71 (br, 64H, CH_2_-N), 3.36 (br, 96H, CH_2_-N), 3.77 (br, 64H, CH_2_-N), 4.80 (br, 8H CH_2_-O), 7.36 (br, 8H, Ar-O), 7.57–8.40 (br, 296H, pyrene, Ar-O), 8.79 (m, 8H, pyrrol), 9.39 (br, 32H, CH=N). ^13^C-NMR (75 MHz, CDCl_3_) δ_C_: 29.4 (CH_2_-NH_2_), 35.7 (CH_2_-N), 37.4 (CH_2_-N), 45.1 (CH_2_-N), 48.7 (CH_2_-N), 50.3 (CH_2_-N), 56.4 (CH_2_-N), 60.4 (CH_2_-N), 67.3 (CH_2_-O), 112.8 (Ar), 119.1 (Ar_ipso_), 121.7 (Ar), 122.57 (Ar), 123,7 (Ar), 124.3 (Ar), 125.5 (Ar), 125.8 (Ar), 126.3 (Ar), 126.9 (Ar), 128.5 (pyrrol), 130.1 (Ar), 130.5 (Ar), 130.6 (Ar), 131.0 (Ar), 135.4 (Ar), 156.6 (CH=N), 168.6 (C=O), MS MALDI TOF *m*/*z*: 10550 (M^+^). Anal. Calcd for C_724_H_578_N_64_O_24_. C 82.36, H 5.52, N 8.49%. Found: C 82.34; H 5.50; N 8.52%.

## 4. Conclusions

Dendrimers bearing 8 and 32 pyrene donor groups have been synthesized. The first and second generations of porphyrin-PAMAM-pyrene dendrimers were obtained in good yields. Fluorescence measurements indicate that, in the first generation dendrimer with 8 pyrene units, excimer emission predominates. For the second-generation dendrimer, the excimer emission decreased and a new peak appeared close to the porphyrin emission peaks, showing an efficient energy transfer from the pyrene units to the core acceptor unit as observed in the photovoltaic response of the first and second-generation compounds, where the second generation molecule exhibited a conversion response larger than first generation. Both compounds produced very regular and homogeneous evaporated films.
